# Limiting of the Innate Immune Response by SF3A-Dependent Control of MyD88 Alternative mRNA Splicing

**DOI:** 10.1371/journal.pgen.1003855

**Published:** 2013-10-24

**Authors:** Lesly De Arras, Scott Alper

**Affiliations:** Integrated Department of Immunology and Integrated Center for Genes, Environment, and Health, National Jewish Health and University of Colorado School of Medicine, Denver, Colorado, United States of America; The University of Texas Health Science Center at Houston, United States of America

## Abstract

Controlling infectious disease without inducing unwanted inflammatory disease requires proper regulation of the innate immune response. Thus, innate immunity needs to be activated when needed during an infection, but must be limited to prevent damage. To accomplish this, negative regulators of innate immunity limit the response. Here we investigate one such negative regulator encoded by an alternative splice form of MyD88. MyD88 mRNA exists in two alternative splice forms: MyD88_L_, a long form that encodes a protein that activates innate immunity by transducing Toll-like receptor (TLR) signals; and a short form that encodes a different protein, MyD88_S_, that inhibits the response. We find that MyD88_S_ levels regulate the extent of inflammatory cytokine production in murine macrophages. MyD88_S_ mRNA levels are regulated by the SF3A and SF3B mRNA splicing complexes, and these mRNA splicing complexes function with TLR signaling to regulate MyD88_S_ production. Thus, the SF3A mRNA splicing complex controls production of a negative regulator of TLR signaling that limits the extent of innate immune activation.

## Author Summary

In response to infection, the body induces the process of inflammation, which is critical to combating the pathogen. However, it also is critical that this inflammatory response be tightly regulated, because overactive or chronically activated inflammation can contribute to a myriad of diseases including sepsis, atherosclerosis, cancer, and Crohn's Disease. Many genes have been identified that either turn on inflammation in response to infection (positive regulators) or turn off the response to ensure that it is limited (negative regulators). Understanding how these negative regulators act may open the door to new therapies to limit inflammation and prevent inflammatory diseases. In the current study, we investigate one such negative regulator called MyD88_S_. We provide a framework to understand how MyD88_S_ is produced, how the body's response to infection alters its production, and how it might be manipulated, which could provide a new means of attack for some of these inflammatory diseases.

## Introduction

The innate immune response plays a key role in fighting infection [1]. Innate immune signaling regulates the process of inflammation, which involves the secretion of cytokines and chemokines and the resulting recruitment and activation of innate and adaptive immune cells [1]. Thus, the activation of the innate immune response is an important positive step in the response to infection. However, an acutely overactive innate immune response or a chronically activated innate immune response can contribute to the pathogenesis of many diseases including sepsis, atherosclerosis, Crohn's disease, and cancer [2]–[5]. Thus it is critical that innate immunity be tightly controlled, activated when necessary and kept inactivate when not. A complex system of receptors and signal transduction molecules control the activation of the innate immune response induced by numerous stimuli. One key class of innate receptors is the Toll-like receptor (TLR) family; different TLRs recognize different pathogen-associated molecular patterns (PAMPs). For example, TLR4 is responsible for the response to lipopolysacharide (LPS) from Gram negative bacteria while TLR2 is responsible for the response to various lipopeptides present in Gram positive bacteria [6], [7].

In addition to the complex regulatory mechanism that controls innate immune activation, there are many negative regulators of innate immunity that limit the response, thereby limiting potential damage due to overactive or chronic inflammation [8]–[13]. Many such negative regulators of innate immunity have been described, including proteins that bind to and inactivate TLR signaling components [8]–[13]; deubiquitinases that inactivate ubiquitinated TLR signaling components [14]; microRNAs that regulate expression of TLR signaling genes [15]; and alternate mRNA splice forms of innate immunity genes, some of which are known to inhibit TLR signaling [16]–[22].

One such alternatively spliced TLR signaling gene is *MyD88* [23]–[26]. MyD88 is a TLR signaling adaptor that acts downstream of most but not all TLRs, and that acts as a positive regulator of innate immunity [6], [7]. In response to stimulation with PAMPs, TLRs dimerize and recruit MyD88. This activates MyD88, which in turn recruits and activates several IL-1 receptor-associated kinases (IRAKs). This complex signal transduction cascade continues, ultimately leading to the production of inflammatory cytokines. MyD88 is encoded by an mRNA with five exons (long form or MyD88_L_). However, a shorter MyD88 mRNA (MyD88_S_) also has been described in both mice and humans [23]–[27]; this mRNA is missing the 135 base pair second exon. Overexpression studies have demonstrated that MyD88_S_ is a negative regulator of TLR signaling that fails to recruit and phosphorylate the IRAKs [23], [24], [26]. MyD88_S_ mRNA levels are drastically increased in monocytes from septic patients suggesting that alterations in MyD88 mRNA splicing could play an important role in human disease [27]. However, thus far, the MyD88_S_ loss of function phenotype has not been reported, and the mechanisms controlling MyD88 alternative mRNA splicing have not been investigated.

Using RNAi screens in mouse macrophage cell lines, we previously identified two members of the SF3A mRNA splicing regulatory complex as regulators of the innate immune response to LPS [28]. The SF3A complex is composed of three proteins (SF3A1, SF3A2, and SF3A3) that interact with the U2 snRNP (small nuclear ribonucleoprotein) [29]. The U2 snRNP interacts with the pre-mRNA branch point near the 3′ splice site in pre-mRNA [30] and facilitates mRNA splicing in conjunction with the rest of the spliceosome [31]–[34]. All three SF3A subunits are required for mRNA splicing [29], [35]–[39].

Here we investigate the innate immune regulatory function of the SF3A mRNA splicing complex and the related splicing factor, SF3B1 which is part of the SF3B complex that functions at a similar step of mRNA splicing to SF3A [40]–[42]. We find that SF3A and SF3B are both required for a robust innate immune response to LPS and other TLR agonists. SF3A and SF3B do so in conjunction with TLR signaling, in part, by regulating the production of the alternate inhibitory splice form MyD88_S_. We also show that changing MyD88_S_ mRNA levels can significantly impact the strength of the innate immune response.

## Results

### The SF3A mRNA splicing complex regulates the innate immune response

**Figure 1:**
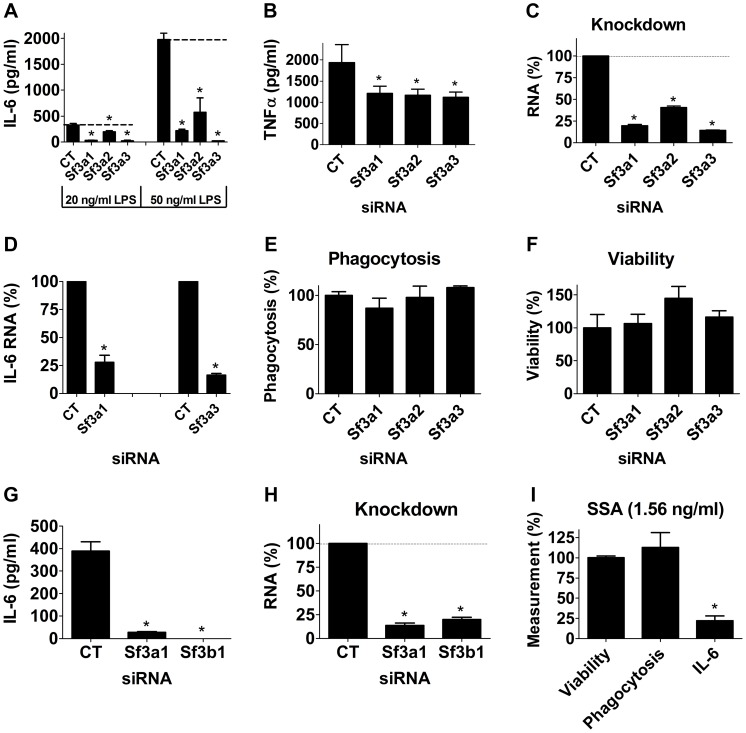
Inhibition of the SF3A complex or SF3B1 diminishes LPS-induced cytokine production. (A–H) The mouse macrophage cell line RAW264.7 was treated with the indicated siRNAs (*Sf3a1*, *Sf3a2*, *Sf3a3*, *Sf3b1*, or control non-targeting siRNA “CT”). The cells were then exposed to LPS for six hours (20 ng/ml except where indicated otherwise) and then the indicated readouts were monitored. (A, G) IL-6 protein production. (B) TNFα protein production. (C, H) Knockdown of each gene induced by the relevant siRNA measured by qPCR. (D) IL-6 mRNA levels. (E) Phagocytosis of FITC-labeled *E. coli* particles. (F) Viability. (I) Cells were treated with 1.56 ng/ml SSA (a pharmacological inhibitor of SF3B1) for six hours; LPS was then added for an additional six hours and the indicated readouts (viability, phagocytosis, IL-6 protein production) were measured and plotted. These three readouts were normalized such that 100% was the value in cells not treated with SSA. Asterisks in this figure and all subsequent figures indicate values that are significantly different from control treatment (p<0.05).

We previously found that inhibition of either *Sf3a1* or *Sf3a2* by RNAi strongly diminished LPS-induced inflammatory cytokine production in either of two mouse macrophage cell lines, RAW264.7 and J774A.1 [28]. To confirm that all three SF3A subunits (SF3A1, SF3A2, and SF3A3) regulated the LPS response, we used siRNAs to inhibit each *Sf3a* subunit in the RAW264.7 macrophage cell line and then monitored LPS-induced production of inflammatory cytokines. Inhibition of each *Sf3a* subunit led to a strong decrease in IL-6 protein production and a more moderate decrease in TNFα protein production (Figure 1A, B). The effect on IL-6 roughly correlated with the extent of knockdown of each subunit as measured by qPCR; siRNA directed against either *Sf3a1* or *Sf3a3* exhibited the strongest gene knockdown (Figure 1C) and the strongest IL-6 phenotypes (Figure 1A). The effect on IL-6 occurred at both the protein (Figure 1A) and mRNA level, as the amount of IL-6 mRNA also was strongly diminished by *Sf3a1* or *Sf3a3* inhibition (Figure 1D). In prior control experiments using RNAi in mouse macrophage cell lines, we found that inhibition of control genes such as TLR4 and MyD88 led to a stronger IL-6 than TNFα phenotype [43], suggesting that the differing extent of inhibition of IL-6 and TNFα production when SF3A is inhibited could be due to differences in the effect of SF3A or could be due to differing sensitivities of the two cytokine promoters to perturbation of signaling.

Because SF3A regulates mRNA splicing, an essential process, it was formally possible that the defects in LPS-induced cytokine production were caused by an overall non-specific decrease in mRNA splicing or fitness caused by *Sf3a* inhibition. However, as outlined below, several experiments argue against this possibility and argue for some specificity. First, other macrophage functions were not affected by *Sf3a* inhibition, including phagocytosis of FITC-labeled *E. coli* particles (Figure 1E). Second, cell viability was not altered by *Sf3a* inhibition (Figure 1F). Third, tests of several other mRNA splicing events indicated that many other mRNA splicing events were not affected when *Sf3a1* was inhibited. For example, we monitored βactin mRNA levels when *Sf3a1* was inhibited by RNAi, comparing expression by qPCR using primers that both annealed to exon 4 or primers that annealed to exons 3 and 4 (thus crossing intron 3); we observed no significant difference between the two primer sets (Figure S1). Similarly, using primers bracketing intron 6 of the *Macf1* gene (another innate immune regulator [28]), we observed no change when *Sf3a1* was inhibited by RNAi (101±13% of control, mean±SEM). Finally, we also monitored alternative mRNA splicing of a TLR regulatory gene known to be alternatively spliced, MD-2. MD-2 exists in two isoforms, MD-2, which along with TLR4 is involved in LPS recognition, and MD-2B, which lacks part of exon 3 and acts as a negative regulator of TLR4 signaling [21]. We observed no significant alteration in production of *MD-2* or *MD-2B* mRNA when *Sf3a1* was inhibited by RNAi (Figure S2).

### The mRNA splicing factor SF3B1 also regulates innate immunity

The fact that inhibition of any of the three *Sf3a* subunits diminished LPS-induced cytokine production suggested that the effect of SF3A on innate immunity was due to its mRNA splicing function. To further test this hypothesis, we used RNAi to inhibit the SF3B1 mRNA splicing factor. RNAi-mediated inhibition of *Sf3b1* also strongly reduced LPS-induced IL-6 production (Figure 1G) and, as expected, diminished *Sf3b1* mRNA levels (Figure 1H). Thus, both the SF3A and SF3B complexes are required for a robust innate immune response to LPS, as inhibition of either diminished that response.

The effects of SF3A1 and SF3B1 were not unique to mouse macrophages; we also found that RNAi-mediated inhibition of *Sf3a1* or *Sf3b1* greatly diminished LPS-induced IL-6 production in human THP1 differentiated macrophages while having only a moderate (SF3B1) or no (SF3A1) effect on viability in these cells (Figure S3).

As a second method to confirm that SF3B1 was required for LPS-induced cytokine production, we used a known pharmacological inhibitor of SF3B1, spliceostatin A (SSA) [44]–[46], to inhibit SF3B1 and then monitored LPS-induced cytokine production. SSA at doses greater than 3 ng/ml diminished overall survival of RAW264.7 cells; these high doses of SSA also diminished the cells' ability to phagocytose FITC-labeled *E. coli* particles (Figure S4). However, at lower SSA doses that did not diminish survival or phagocytic ability, treatment of RAW264.7 macrophages with SSA led to a profound decrease in LPS-induced IL-6 production (Figure 1I, S4). Thus, inhibition of SF3B1 by RNAi or with a pharmacological agent strongly inhibited the innate immune response to LPS.

**Figure 2:**
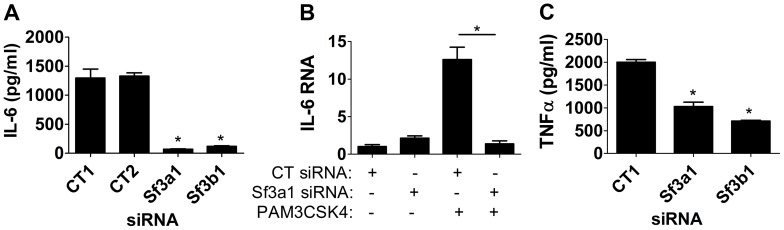
Inhibition of SF3A1 or SF3B1 diminishes PAM3CSK4-induced inflammatory cytokine production. Cells were transfected with the indicated siRNAs (either *Sf3a1*, *Sf3b1*, or either of two different control non-targetting siRNAs) and treated with PAM3CSK4 where indicated (B) or in all cases (A,C). Subsequently IL-6 protein (A), IL-6 mRNA (B), or TNFα protein (C) levels were monitored. In panel B, RNA levels of 1 are defined as IL-6 mRNA levels in the presence of control siRNA treatment in the absence of PAM3CSK4.

To determine if these mRNA splicing regulators affected other innate immune stimuli, we also monitored the effect of SF3A1 and SF3B1 when cells were stimulated with the TLR2/1 agonist PAM3CSK4 [47]. Inhibition of either *Sf3a1* or *Sf3b1* by RNAi led to a strong decrease in IL-6 protein, IL-6 mRNA, and TNFα levels (Figure 2). The effect of SF3A1 and SF3B1 did not extent to all stimuli, however, as inhibition of these genes by RNAi did not inhibit the response to the TLR3 agonists poly(I:C) (Figure S5) or poly(A:U) (data not shown) [we monitored production of TNFα rather than IL-6 in these assays because poly(I:C) induced little IL-6].

TLR4 uses two signaling adaptors, MyD88 and TRIF, to control the response to LPS. SF3A1 and SF3B1 likely regulate MyD88 signaling, because they affect LPS and PAM3CSK4-induced production of IL-6 and TNFα. To determine if SF3B1 affects the TRIF-dependent arm of the TLR4 pathway, we monitored production of IFNβ by qPCR when SF3B1 was inhibited by SSA; we found that SSA treatment diminished both IL-6 and IFNβ production (Figure S6), indicating that SF3B1 has both MyD88-dependent and MyD88-independent effects downstream of TLR4.

### SF3A1 acts upstream in the TLR4 signaling pathway

**Figure 3:**
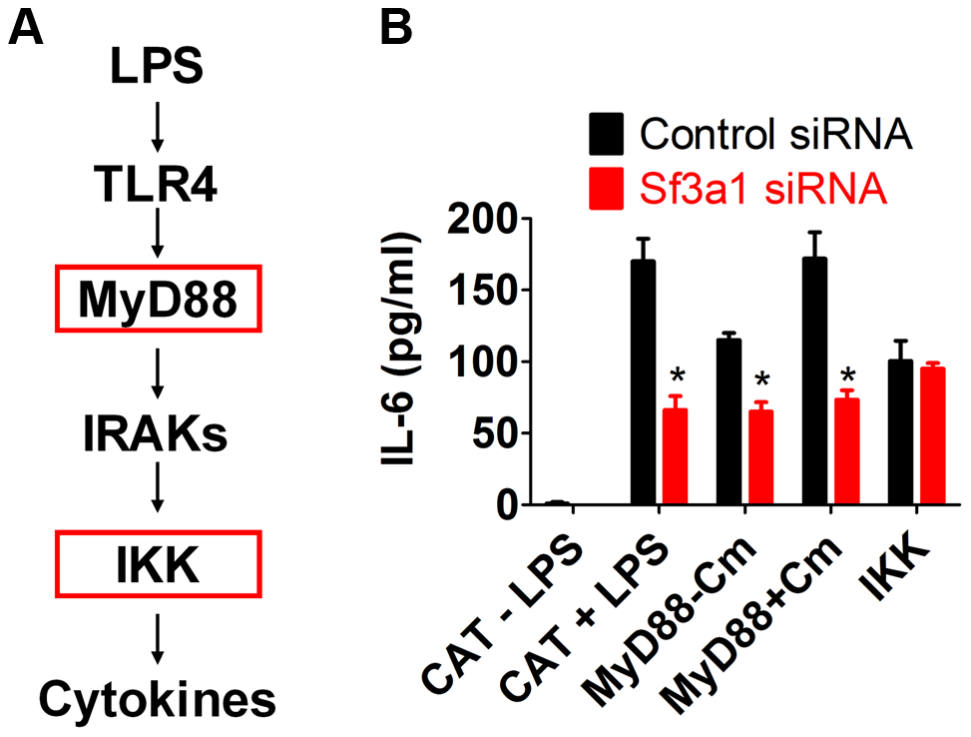
SF3A1 exerts its effects on innate immunity downstream of MyD88 and upstream of IKK. (A) Schematic that depicts the relevant proteins in the TLR signaling cascade; for simplicity, many proteins are not depicted. Red boxes indicates activated constructs tested in this assay. (B) RAW264.7 cells were transfected both with the indicated siRNA (*Sf3a1* siRNA shown in red, control non-targetting siRNA shown in black) and plasmids overexpressing the indicated constructs (inducibly active MyD88-gyrB, constitutively active IKK, or negative control CAT). Cells were also treated with LPS or Coumermycin A (Cm) as indicated. The graph depicts IL-6 protein production.

To investigate the relationship between SF3A1 and TLR signaling further, we used RNAi to inhibit *Sf3a1* in cells that expressed activated TLR signaling components (Figure 3A). These activated constructs included a constitutively active IKK construct containing two mutations in the active site [IKK-2S177ES181E [48]] and an inducibly active MyD88 construct [49]. MyD88 was rendered inducibly active in a construct in which full length MyD88 is fused to the effector domain of subunit B of *E. coli* DNA gyrase [49]; treatment of cells with the antibiotic coumermycin [49] leads to dimerization of DNA gyrase B and thus dimerization and activation of MyD88. As a negative control, we also inhibited *Sf3a1* by RNAi in macrophages expressing chloramphenicol acetyltransferase (CAT), which should not alter innate immunity.

As expected, cells expressing the negative control protein CAT produced IL-6 in the presence but not the absence of LPS, and further inhibition of *Sf3a1* in these cells led to a strong decrease in IL-6 production (Figure 3B). Cells overexpressing the MyD88-gyrB fusion produced IL-6 in the absence of LPS, and this was enhanced by the addition of coumermycin, as expected (Figure 3B). The MyD88-gyrB fusion expresses full length MyD88 protein. MyD88 mRNA levels were 162±24 (mean±SEM)-fold higher than normal (determined by qPCR) in cells overexpressing MyD88-gyrB, and this high level of full length MyD88 may be able to activate signaling even in the absence of the dimerizing agent. Nevertheless, *Sf3a1* siRNA diminished IL-6 production when MyD88-gyrB was overexpressed (Figure 3B). However, *Sf3a1* inhibition did not prevent IL-6 production induced by overexpression of the constitutively activated IKK construct (Figure 3B). These data are consistent with SF3A1 exerting its effect on TLR signaling pathways, acting downstream of MyD88 and upstream of IKK.

### Inhibition of SF3A1 or SF3B1 enhances production of MyD88_S_

**Figure 4:**
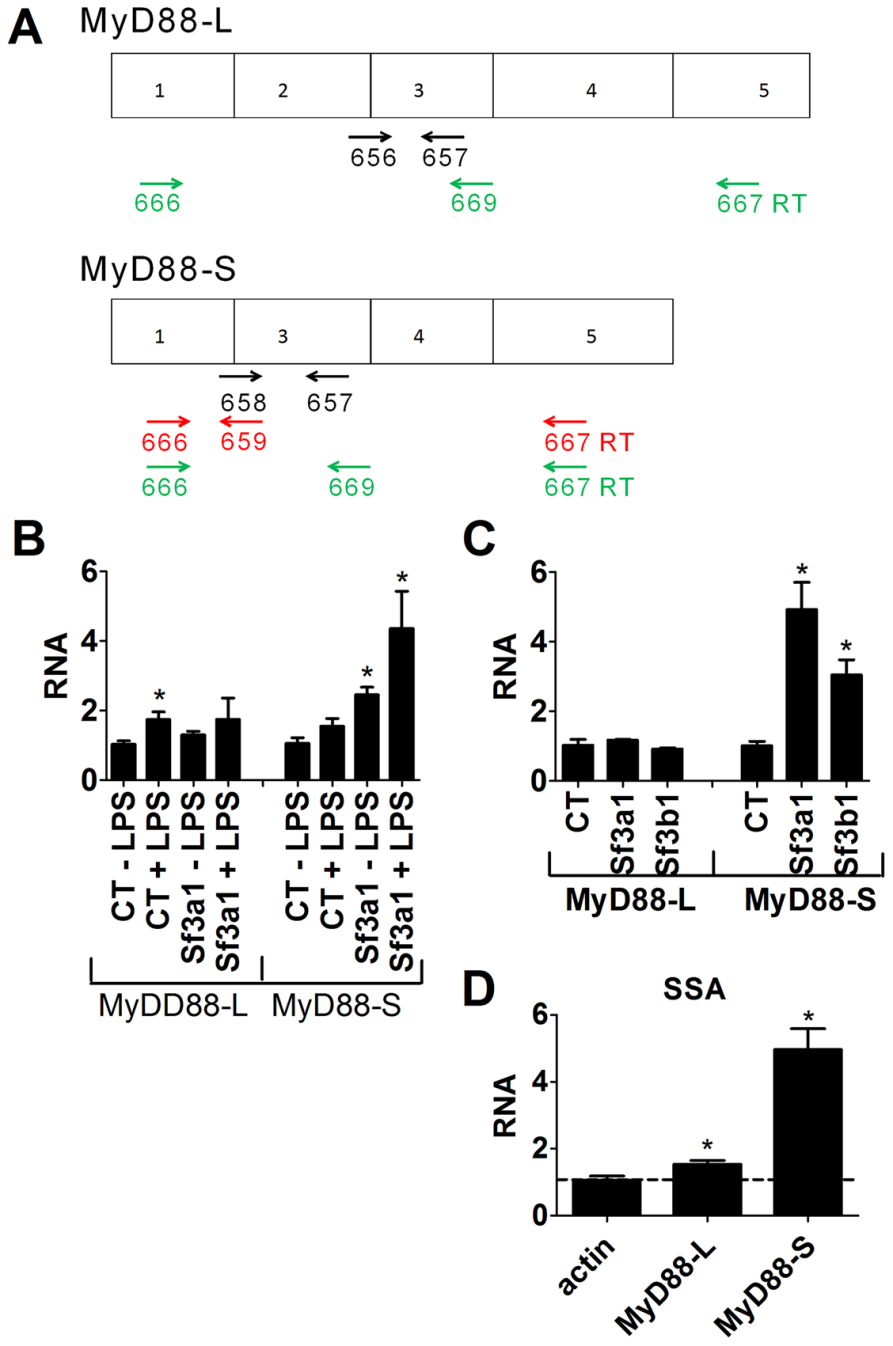
qPCR assay demonstrates that inhibition of SF3A1 or SF3B1 enhances production of MyD88_S_ mRNA. (A) Depicts the MyD88_L_ and MyD88_S_ alternate mRNA splice forms as well as the location of the primers used to monitor the production of *MyD88_L_* or *MyD88_S_*. qPCR primers to detect either *MyD88_L_* or *MyD88_S_* are shown in black. Reverse transcription primers to detect *MyD88_S_* are shown in red. Reverse transcription primers to detect both *MyD88_L_* and *MyD88_S_* simultaneously are shown in green. (B) The indicated siRNAs were transfected into RAW264.7 cells, the cells were stimulated with LPS as indicated, and *MyD88_L_* and *MyD88_S_* mRNA levels were monitored by qPCR. (C) Cells were transfected with the indicated siRNAs, were stimulated with LPS, and *MyD88_L_* and *MyD88_S_* mRNA levels were monitored by qPCR. (D) RAW264.7 cells were treated with 1.1 ng/ml SSA for six hours, were subsequently exposed to LPS for six hours (in the presence of SSA), and βactin, *MyD88_L_* and *MyD88_S_* mRNA levels were monitored by qPCR (normalized relative to βactin primers that cross intron 3). RNA of 1 is defined as *MyD88_L_* or *MyD88_S_* mRNA levels in the presence of the control non-targetting siRNA in the absence of LPS (B) or in the presence of LPS (C and D).

Because multiple SF3A subunits and SF3B1 all regulate LPS-induced cytokine production, we hypothesized that these mRNA splicing factors were regulating the splicing of a TLR-regulatory gene(s). Moreover, this gene likely acts downstream of MyD88 and upstream of IKK. One candidate that fits these data is MyD88 itself, as the alternate splice form MyD88_S_ inhibits IRAK activation downstream of MyD88 and upstream of IKK [23], [24], [26]. We therefore chose to monitor MyD88 mRNA splicing by qPCR using primers that can distinguish between the five exon *MyD88_L_* splice form and the four exon *MyD88_S_* splice form (Figure 4A). For the qPCR studies, we detected *MyD88_L_* using a primer that spanned exons 2 and 3 and a second primer in exon 3 (Figure 4A). We detected *MyD88_S_* using a primer that spanned exons 1 and 3 and a second primer in exon 3 (Figure 4A). Inhibition of *Sf3a1* in either the presence or absence of LPS did not alter *MyD88_L_* mRNA levels substantially but did increase the *MyD88_S_* shorter splice form (Figure 4B). Both LPS and *Sf3a1* inhibition contributed to this increase in *MyD88s* mRNA (Figure 4B).

To test if SF3B1, like SF3A1, regulated MyD88_S_ mRNA levels, we inhibited SF3B1 using either RNAi or SSA treatment and monitored *MyD88_L_* and *MyD88_S_* mRNA by qPCR. Inhibition of *Sf3b1* by RNAi, like inhibition of *Sf3a1*, did not significantly alter *MyD88_L_* mRNA levels in the presence of LPS but did significantly increase *MyD88_S_* mRNA levels in the presence of LPS (Figure 4C). Similarly, inhibition of SF3B1 by SSA had at most a moderate effect on *MyD88_L_* mRNA levels while leading to a substantial increase in *MyD88_S_* mRNA levels (Figure 4D). As a control to confirm that not all siRNA treatments altered MyD88 mRNA splicing, we found that RNAi-mediated inhibition of the 26S proteasome subunit Psmd3 greatly diminished LPS-induced IL-6 production [43] (Figure S7A) without significantly altering production of MyD88_L_ or MyD88_S_ (Figure S7B,C).

The qPCR data indicated that one of the wild type functions of SF3A1 and SF3B1 is to inhibit production of MyD88_S_, as MyD88_S_ levels are increased when either mRNA splicing factor is inhibited. To confirm these MyD88 mRNA qPCR data, we monitored *MyD88_L_* and *MyD88_S_* mRNA levels using semi-quantitative reverse transcription-PCR followed by agarose gel elecrophoresis. For these experiments, we used two sets of primers, one set designed to specifically monitor *MyD88_S_* levels and one set designed to monitor both *MyD88_L_* and *MyD88_S_* simultaneously (Figure 4A). For the *MyD88_S_*-specific primers, we used a reverse primer that spanned exons 3 to 1 and a forward primer that annealed to exon 1 (Figure 4A); these primers generate a 124 bp product when *MyD88_S_* is present. For primers that amplify both *MyD88_L_* and *MyD88_S_*, we used primers that annealed to exons 1 and 3 (Figure 4A); these generate a 370 bp band corresponding to *MyD88_L_* and a 235 bp band corresponding to *MyD88_S_*.

**Figure 5:**
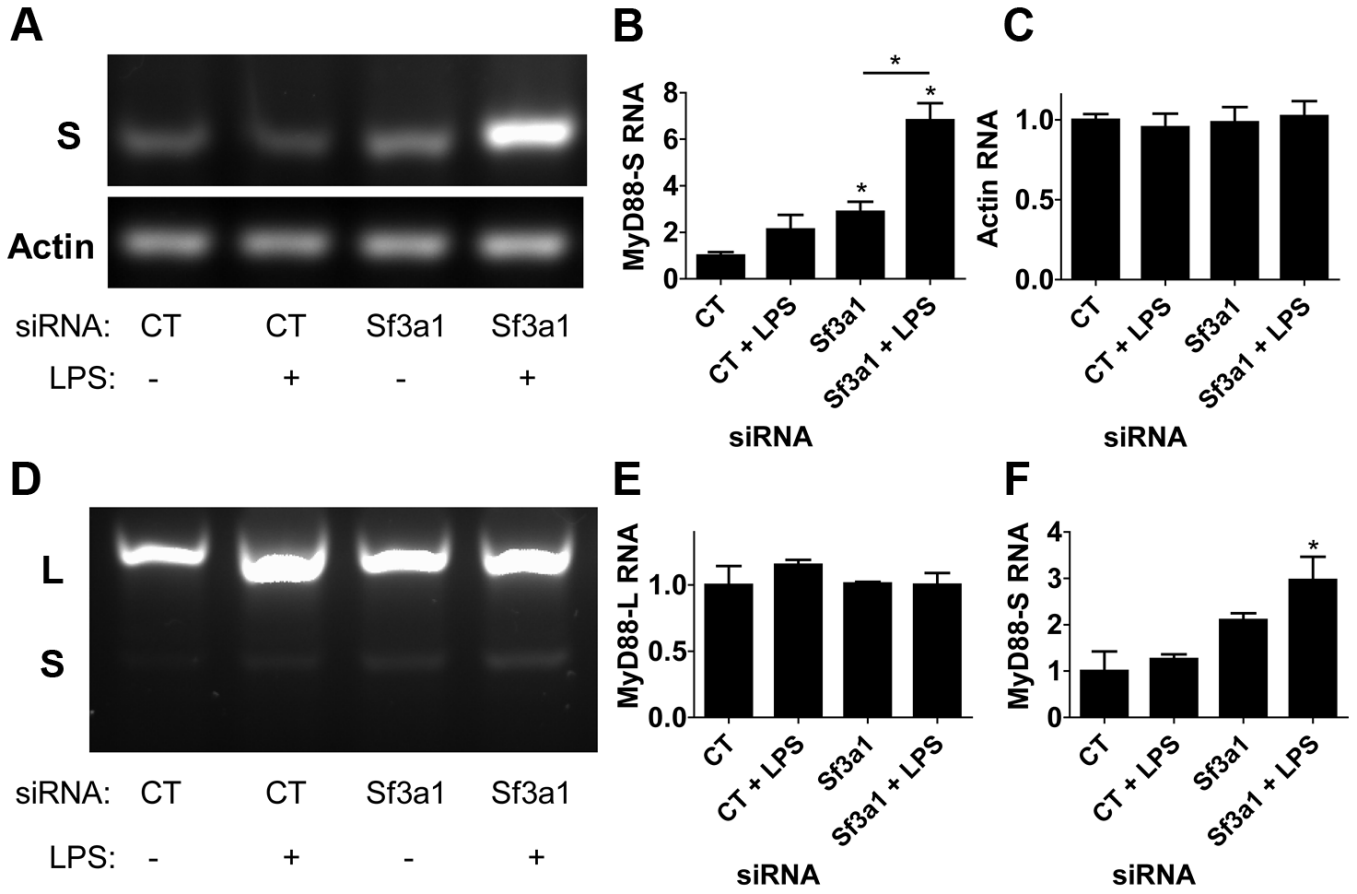
RT-PCR assay demonstrates that inhibition of SF3A1 enhances production of MyD88_S_ mRNA. Cells were exposed to the indicated siRNAs and LPS where indicated, the cells were then lysed, RNA was purified, the RNA was reverse transcribed, and the resulting cDNA was subjected to PCR using primers outlined in Figure 4A. The resulting PCR products were then subjected to agarose gel electrophoresis. (A) Representative image of amplification of an mRNA product specific to *MyD88_S_* (S in top gel) or mRNA product specific to βactin (bottom gel). Quantitation of the bands in this gel and two other gels are depicted in (B) *MyD88_S_* and (C) βactin. (D) depicts a representative image of RT-PCR amplification using primers that bracket MyD88 exon 2 and that therefore amplify products from both *MyD88_L_* and *MyD88_S_* (indicated by L and S to left of panel). Quantitation of the bands in this gel and two other gels are depicted in (E) *MyD88_L_* and (F) *MyD88_S_*. A longer exposure of the gel in panel D is displayed in Figure S8.

Using the *MyD88_S_*-specific RT-PCR primers (Figure 4A), we found that treatment of cells with either LPS or *Sf3a1* siRNA alone had a fairly moderate effect on *MyD88_S_* mRNA levels (agarose gel in Figure 5A, quantitation from three independent experiments in Figure 5B). However, cells treated with both LPS and *Sf3a1* siRNA exhibited a substantial increase in *MyD88_S_* mRNA (Figure 5A, B). As a control, we found that these treatments did not alter production of βactin (Figure 5A, C).

Using the reverse transcription primers that amplified both *MyD88_L_* and *MyD88_S_* simultaneously (Figure 4A), we were able to visualize *MyD88_L_* and *MyD88_S_* (Figure 5D). Consistent with the qPCR data, treatment of cells with LPS and *Sf3a1* siRNA either alone or together did not significantly alter *MyD88_L_* mRNA levels (agarose gel in Figure 5D, quantitation from three independent experiments in Figure 5E). However, treatment of cells with LPS and *Sf3a1* siRNA did significantly enhance *MyD88_S_* mRNA levels (Figure 5D, F and S8).

### Changes in MyD88_S_ levels can have a significant impact on LPS-induced cytokine production

The RT-PCR experiments using primer sets that amplified both *MyD88_L_* and *MyD88_S_* allowed us to estimate the ratio of *MyD88_L_* to *MyD88_S_*. In the absence of treatment, this ratio was roughly 20∶1; in the presence of LPS and *Sf3a1* siRNA, the *MyD88_L_* to *MyD88_S_* ratio increased significantly to approximately 5∶1. This raised the question of whether this change in MyD88_S_ was sufficient to overcome the greater MyD88_L_ level and alter innate immunity significantly. To test this directly, we engineered a siRNA that spanned the exon 1–3 boundary in *MyD88_S_* (and would thus not be present in *MyD88_L_*) with the goal of designing an siRNA that specifically inhibits *MyD88_S_* but not *MyD88_L_*. We tested two siRNAs in this fashion that differed in their start location by only one base; one did not affect *MyD88_S_* levels significantly (not shown) but the other did, as described below.

**Figure 6:**
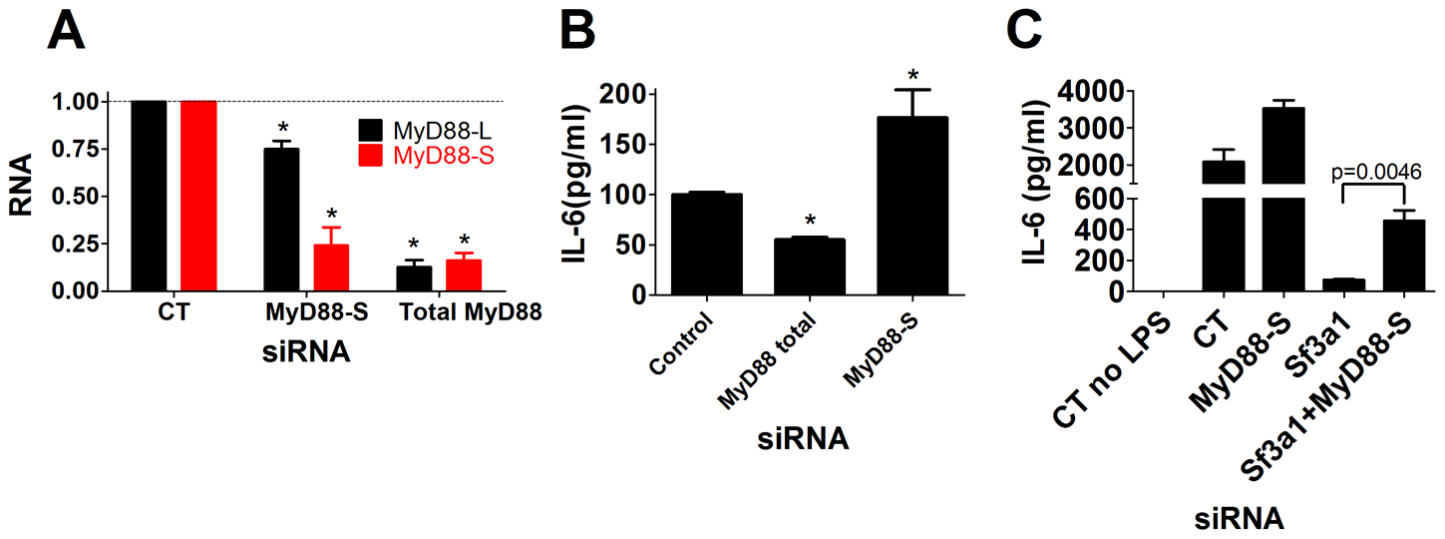
Specific inhibition of MyD88_S_ enhances LPS-induced cytokine production and partially rescues the innate immune defect caused by SF3A1 RNAi. The indicated siRNAs were transfected into the RAW264.7 cell line, cells were stimulated with LPS, and either the extent of gene knockdown (*MyD88_L_* or *MyD88_S_* in panel A) or IL-6 production (B,C) are depicted.

Treatment of macrophages with the *MyD88_S_*-specific siRNA led to a roughly 75% decrease in *MyD88_S_* mRNA levels while having a more moderate effect on *MyD88_L_* mRNA (Figure 6A), indicating some degree of efficacy and specificity. As a control, treatment of cells with a pool of four siRNAs targeting total *MyD88* led to an 80% decrease in both *MyD88_L_* and *MyD88_S_* levels, as expected (Figure 6A). We therefore used these siRNAs to monitor the effect of changes in total *MyD88* or *MyD88_S_* levels on LPS-induced cytokine production. Inhibition of total *MyD88* (*MyD88_L_*+*MyD88_S_*) decreased LPS-induced IL-6 production (Figure 6B), as expected. In contrast, inhibition of *MyD88_S_* using the *MyD88_S_*-specific siRNA led to significantly increased LPS-induced IL-6 production (Figure 6B). This near doubling in cytokine production was impressive as the knockdown of *MyD88_S_* using this siRNA was incomplete and there was also some effect on *MyD88_L_*, both of which could allow us to underestimate the true scope of the effect of MyD88_S_.

### MyD88_S_ mediates some of the effect of *Sf3a1* inhibition on innate immunity

It was possible that all of the effects of SF3A1 on innate immunity could be mediated by changes in MyD88_S_ levels; alternatively, it was possible that SF3A1 affects production of several regulators of TLR signaling including MyD88_S_. To distinguish between these possibilities, we treated cells with two siRNAs simultaneously: siRNA targeting *Sf3a1* and siRNA targeting *MyD88_S_*. Inhibition of *Sf3a1*, as described above, strongly diminished LPS-induced IL-6 production (Figure 6C). Inhibition of *Sf3a1* and *MyD88_S_* simultaneously partially restored IL-6 production (Figure 6C). Thus, at least some of the effect of *Sf3a1* inhibition is mediated by increases in *MyD88_S_* levels. As a control, qPCR used to monitor gene knockdown demonstrated that the various siRNA treatments were acting as expected (Figure S9). However, the small effect of the *MyD88_S_*-specific siRNA on *MyD88_L_* makes it difficult to determine with certainty if this incomplete rescue is due to limitations of the RNAi experiment or because other TLR regulators are also affected by SF3A1. Regardless, it is clear that changes in *MyD88_S_* levels account for at least part of the effect of SF3A1 on innate immunity.

## Discussion

### MyD88_S_ modulates the extent of the innate immune response

MyD88_S_ has been observed in multiple mouse and human cells, cell lines, and tissues [23]–[25], [27], [50], [51]. Prior studies investigating MyD88_S_ used overexpression or reintroduction strategies to demonstrate that MyD88_S_ was a TLR signaling inhibitor that functioned by inhibiting IRAK phosphorylation [23], [24], [26]. By developing a siRNA that targets the unique splice junction in MyD88_S_, we have now been able to demonstrate in a MyD88_S_ loss-of-function experiment that MyD88_S_ is, as demonstrated in the published overexpression studies, an inhibitor of the innate immune response. Moreover, we show that while there is far more MyD88_L_ than MyD88_S_ in macrophages, changes in the amount of the minor MyD88_S_ splice form are sufficient to overcome the much larger pool of MyD88_L_ and affect inflammatory cytokine production. This is consistent with published overexpression data that indicate that MyD88_S_ is able to inhibit a large pool of MyD88_L_ [24].

### The SF3A/SF3B mRNA splicing complexes regulate the innate immune response in part by regulating MyD88_S_ levels

We have found that inhibition of the SF3A or SF3B complexes by RNAi or with a pharmacological agent leads to a strong decrease in production of the pro-inflammatory cytokine IL-6 and a more moderate decrease in production of the pro-inflammatory cytokine TNFα without affecting macrophage viability or phagocytosis. Other reports indicate that RNAi-mediated knockdown of *Sf3a* subunits can affect cell survival in HeLa cells [38]; moreover, a knockout in *Sf3b1* in mice is lethal, although heterozygous *Sf3b1* mice develop largely normally [52]. The difference in our data may be due to incomplete but still very strong knockdown in macrophages or other cell-type-specific differences.

**Figure 7:**
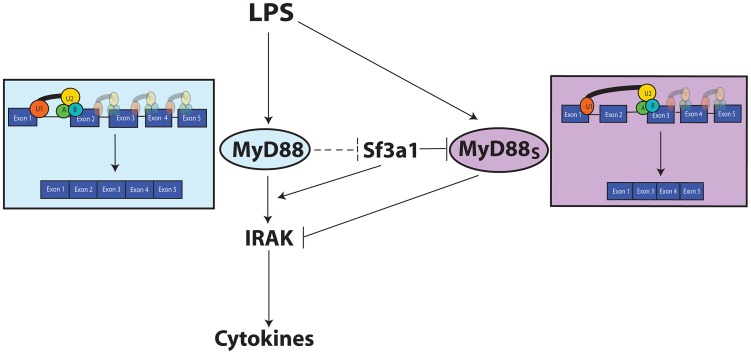
Model depicting the relationship between TLR signaling, MyD88, SF3A1, and MyD88_S_. Both SF3a1 and LPS can alter production of MyD88_S_, which is known to inhibit TLR signaling at the level of the IRAKs. When SF3A levels or activity are diminished, MyD88_S_ levels are enhanced by skipping of MyD88 exon 2. The dashed inhibition line in the figure depicts one possible scenario by which TLR signaling could regulate MyD88_S_ levels, as outlined in the Discussion. The figure also indicates that SF3a likely has other effects on innate immunity. For simplicity, most known TLR signaling proteins are not depicted.

One of the innate immune targets of SF3A and SF3B is production of the alternate splice form of MyD88, MyD88_S_ (Figure 7). The spliceosome is a large multi-subunit protein and RNA complex that facilitates intron removal in two catalytic steps. First, the 5′ splice site is cleaved resulting in the formation of a lariat structure. Second, the 3′ splice site is cleaved and the exons are ligated together [29], [32], [33]. The SF3A and SF3B complexes in conjunction with the U2 snRNP bind to the branch site near the 3′ splice site to facilitate mRNA splicing [30], [35]–[42], . These mRNA splicing complexes play an important role in proper 3′ splice site recognition; this is evidenced by the frequent observation of altered mRNA splicing observed when SF3B1 is inhibited or mutated [44], [54]–[56]; in many cases, inappropriate exon skipping is observed. This phenomenon of altered splicing and exon skipping is also consistent with the effect of SF3A1 or SF3B1 inhibition on MyD88_S_ production; MyD88_S_ is produced if the 3′ splice site at the end of intron 1 is skipped and the 3′ splice site at the end of intron 2 is used instead (Figure 7). It is intriguing to speculate that the MyD88 splice site choice evolved to be exquisitely sensitive to cellular conditions because of its functional significance, and may be a key point of regulation to limit inflammation.

### The SF3A/SF3B mRNA splicing complexes likely also affect other genes to modulate innate immunity

While our data indicate the importance of MyD88_S_ in mediating the innate immune effects of SF3A/SF3B, our data also suggest that other regulator(s) of TLR signaling also are affected by these mRNA splicing regulators. Inhibition of MyD88_S_ using our *MyD88_S_*-specific siRNA is only able to partially rescue the innate immune defect caused by inhibition of *Sf3a1*. It is unclear if this incomplete rescue is due to limitations of the RNAi experiment or because another gene(s) are also regulated by SF3A1, although we favor the latter possibility. Several other pieces of data argue that SF3A/SF3B are affecting other genes besides MyD88 to regulate innate immunity, including the observation that SF3B1 inhibition can affect LPS-induced IFNβ production. Moreover, we did not observe significant inhibition of IRAK1 activation when SF3a1 is inhibited (data not shown); while the regulation of IRAK1 by MyD88_S_ has not been studied in a loss-of-function context, these data also are consistent with the possibility that SF3a1 regulates innate immunity using both MyD88_S_-dependent and independent means.

### mRNA splicing factors, MyD88_S_, and human disease

By inhibiting MyD88_S_ using a *MyD88_S_*-specific siRNA, we demonstrated a significant increase in inflammatory cytokine production. Even a moderate increase in inflammation could have a significant impact on disease. Adib-Conquy *et al.* [27] observed that there was a roughly 10-fold increase in MyD88_S_ levels in monocytes from patients with sepsis, which could explain the immunosuppressed phenotype of these cells. The SF3B1 and SF3A1 mRNA splicing factors have also been implicated in the pathogenesis of numerous hematologic malignancies. In particular, *Sf3b1* mutations are prevalent in a wide range of hematologic malignancies, associated with 5% of acute myeloid leukemia cases, 10–15% of chronic lymphocytic leukemia cases, and 60–80% of the myelodysplastic syndrome subtype Refractory Anemia with Ring Sideroblasts (MDS-RARS) [57]–[68]. Chronic inflammation has been implicated in the pathogenesis of many solid and hematologic malignancies [4], [69]–[73]; conceivably, altered innate immunity signaling could be one of the factors involved in the pathogenesis of these malignancies.

### The relationship between TLR signaling, MyD88, and SF3A1 function

MyD88_S_ inhibits TLR signaling at the level of the IRAK kinases [23], [24], [26] (Figure 7). We have now demonstrated that the SF3A and SF3B complexes inhibit production of this alternative MyD88_S_ splice form (Figure 7). Prolonged LPS exposure is reported to enhance MyD88_S_ production [24], and our data also indicates that LPS can enhance MyD88_S_ levels (Figure 7). It is possible that SF3A1 could act either in parallel to or downstream of TLR signaling to control MyD88_S_ production.

How might the TLR pathway interact with the SF3A mRNA splicing complex? Some intriguing published protein interaction studies raised the possibility that MyD88 itself could directly regulate the SF3A complex to modulate its own mRNA splicing. Human SF3A3 has been reported to bind to MyD88 in liver cells using an immunoprecipitation-mass spectrometry assay [74]. The *C. elegans* SF3A1 ortholog has been reported to bind to the sole *C. elegans* MyD88 family member TIR-1 in a yeast 2 hybrid assay [75]. TIR-1 is most homologous to mammalian SARM, a negative regulator of mammalian innate immunity [76], [77], but functionally behaves most like MyD88, in that it is required for host defense to many classes of pathogens and acts upstream of the p38 MAPK pathway [78]–[80]. Finally, *Drosophila* SF1 (another mRNA splicing factor that interacts with the U2 snRNP) has been reported to interact with *Drosophila* MyD88 in a yeast two hybrid assay [81]. While the SF3A complex functions in the nucleus to control mRNA splicing, it is assembled in the cytoplasm [82], [83] and could be available for modification by MyD88, at least transiently. Future studies will be needed to determine the biological significance of this interaction. However, these data raise the possibility of a MyD88-mediated negative feedback loop that ensures that innate immunity is self limiting (Figure 7); this could be relevant to sepsis, cancer, and the myriad of other diseases with an inflammatory component [84].

## Materials and Methods

### RNAi experiments

RNAi using RAW264.7 cells was performed largely as described [43]. In brief, pools of siRNA duplexes (Dharmacon) targeting the indicated genes were transfected into the mouse macrophage cell line RAW264.7 using the Amaxa 96-well nucleofector shuttle. Cells were then either plated in 100,000 cells per well in 96-well format for subsequent ELISA assays or 200,000 cells/well in 24-well format for subsequent qPCR analysis. The *MyD88_S_*-specific siRNA sequence was 5′-GAAGTCGCGCATCGGACAA-3′. We also tested a second siRNA that was shifted one base pair 3′; this siRNA did not significantly inhibit *MyD88_S_* mRNA levels. Negative control siRNAs were Dharmacon's non-targeting siRNA pool #1 and in some cases non-targetting siRNA #1. Twenty-four hours after siRNA transfection, cells were stimulated with the indicated PAMPs for six hours. Doses used were 20 ng/ml LPS (List Biological Labs), 1.5 µg/ml PAM3CSK4 (Invivogen), or 6 µg/ml poly(I:C) (Invivogen). Several different assays were then performed. In some cases, cell supernatants were collected and cytokine production was monitored by ELISA (R&D Biosystems). Viability of the remaining adherent cells was monitored using fluorescein diacetate, which is cleaved into a fluorescent form in intact live cells [85]. In other experiments, RNAi-treated cells were analyzed for their ability to phagocytose FITC-labeled *E. coli* particles using the Vybrant Phagocytosis assay kit (Molecular Probes). In other experiments, RNAi-treated cells were lysed in RLT buffer (Qiagen) and used to prepare RNA for qPCR or RT-PCR.

In the experiments using two different siRNAs at the same time in RAW264.7 cells, the siRNAs were transfected simultaneously with an equal volume of each. In these dual transfection experiments, when only a single siRNA was used, the volume of siRNA was made up with an equal volume of Dharmacon non-targetting siRNA pool #1.

RNAi in the human THP1 cell line was performed by first transfecting pools of siRNAs targeting either SF3A1 or SF3B1 (and control non-targeting siRNA pool) using the Amaxa 96-well nucleofector shuttle. Cells were then plated at 200,000 cells per well in 96-well format. 8 hours later, 50 ng/ml phorbol 12-myristate 13-acetate (PMA) was added. 24 hours later, the cells were exposed to 50 ng/ml LPS for six hours, and then IL-6 production and viability were monitored.

### Treatment of cells with both siRNA and overexpression plasmids

In the experiments in which both plasmids and siRNAs were delivered to RAW264.7 macrophages, cells were first transfected in 12 well-format with the indicated plasmids (1.6 µg each) using 0.7% (v/v) Fugene HD (Roche) for transfection. 24 hours later, cells were transfected with siRNA in 96 well-format using the Amaxa system. 24 hours later, cells were exposed to either of 0.1 µM Coumermycin A (Sigma) or LPS for six hours as indicated, and then cytokine production was monitored by ELISA. Transfection efficiency using Fugene HD was roughly 40% (determined using a plasmid expressing GFP and fluorescence microscopy) and was not affected by the different siRNA treatments.

### qPCR and RT-PCR to monitor mRNA levels

RNA for analysis was prepared by lysing cells in RLT buffer and using the Qiagen RNAeasy kit for RNA purification. qPCR was performed on an ABI 7900 using the Qiagen Quantitect SYBR-green assay kit. Data was normalized using βactin as a control. Because we were monitoring the effect of known mRNA splicing regulators, we tested primers that were entirely internal to exon 4 and primers that crossed intron 3; both gave similar results in all experiments.

mRNA levels were also analyzed in semi-quantitative fashion using RT-PCR followed by agarose gel electrophoresis. 500 ng total RNA prepared as described above was first subjected to reverse transcription using Superscript III reverse transcriptase (Invitrogen). The reverse transcribed cDNA was then subjected to PCR using Taq DNA polymerase (Invitrogen), PCR products were subjected to electrophoresis in 2% agarose gels, and images were captured using a UV box and a CCD camera.

The sequences of all primers are listed in Table S1.

We were unable to visualize MyD88_S_ protein by western blot using three different commercial sources of MyD88 antisera; presumably, this is reflective of the large ratio of MyD88_L_∶MyD88_S_ that we observed using RT-PCR (Figure 5D). This also is consistent with prior published studies in which MyD88_S_ protein could be monitored by western blot in RAW264.7 cells in which the entire pool of MyD88_L_ is artificially converted to MyD88_S_ using antisense oligonucleotides [86], but not in otherwise wild-type cells [24].

### Use of Spliceostatin A (SSA) to inhibit SF3B1

Cells were treated with the indicated doses of SSA (diluted in methanol from a 100 µg/ml stock in methanol) for six hours. Methanol was kept at less than <0.5% total volume, was matched in control cells, and didn't alter cytokine production or viability. After the six hour SSA exposure, LPS was added to the media containing SSA for an additional six hours. Then viability, phagocytosis, cytokine production, and various mRNA levels, were analyzed as outlined above.

### Statistical analyses

All data are from a minimum of three biological replicates. Data were graphed and analyzed using Graphpad Prism 5. Statistically significant differences in all experiments were considered p<0.05 and were determined using t-tests. Bands on agarose gels were quantified using Image J [87] and subsequently analyzed for significance in Graphpad Prism 5. While we only display representative images of agarose gels from one experiment, the analyzed data and statistics are from three independent experiments.

## Supporting Information

Figure S1 Inhibition of SF3A1 does not alter excision of βactin intron 3. RAW264.7 cells were transfected with either *Sf3a1* siRNA or control non-targeting siRNA (CT) and were subsequently stimulated with LPS (20 ng/ml for 6 hr) or not as indicated. Cells were then lysed and qPCR was used to monitor βactin mRNA levels. The figure depicts mRNA levels for primers that both annealed to exon 4 normalized relative to primers that annealed to exons 3 and 4 and therefore span intron 3. No significant difference was observed between the two primer sets.
(PDF)

Figure S2 Inhibition of SF3A1 does not alter the alternative splicing of *MD-2* mRNA. RAW264.7 cells were transfected with either *SF3a1* siRNA or control non-targeting siRNA (CT) and were subsequently stimulated with LPS (20 ng/ml for 6 hr) or not as indicated. Cells were then lysed and qPCR was used to monitor production of *MD-2* or *MD-2B* mRNA. No significant difference was observed for either isoform.
(PDF)

Figure S3 Inhibition of SF3A1 or SF3B1 in differentiated human THP1 macrophages diminishes LPS-induced IL-6 production. The human monocyte cell line THP1 was transfected with the indicated siRNAs (*Sf3a1*, *Sf3b1*, or control non-targeting siRNA “CT”). The cells were then differentiated with PMA, exposed to LPS for six hours (50 ng/ml), and then IL-6 (panel A) and viability (panel B) were monitored. Asterisks indicate values that are significantly different from control treatment (p<0.05).
(PDF)

Figure S4 Treatment of cells with spliceostatin A (SSA) inhibits LPS-induced IL-6 production. The figure depicts a dose-response titration of SSA at the indicated doses. Cells were treated with SSA for six hours, were subsequently exposed to 20 ng/ml LPS for an additional six hours (in the presence of SSA), and then the cells were monitored for either viability, phagocytosis of FITC-labeled *E. coli* particles, or production of IL-6. All values are normalized relative to control cells not treated with SSA. At high doses, SSA diminishes all three readouts. However, at lower doses, SSA inhibits IL-6 production without significantly affecting viability or phagocytosis.
(PDF)

Figure S5 Inhibition of SF3A1 or SF3B1 weakens the response to TLR4 and TLR2/1 agonists but not the response to TLR3 agonists. RAW264.7 cells were transfected with the indicated siRNAs (SF3a1, SF3b1, or control nontargeting siRNA “CT”). The cells were then stimulated with either 20 ng/ml LPS, 1.5 µg/ml PAM3CSK4, or 6 µg/ml poly(I:C) for six hours, and then TNFα production was monitored by ELISA. Asterisks indicate values that are significantly different from control treatment (p<0.05).
(PDF)

Figure S6 SSA treatment inhibits LPS-induced production of IL-6 and IFNβ. RAW264.7 cells were treated with 1.56 ng/ml SSA for 12 hours. The cells were then stimulated with 20 ng/ml LPS for six hours where indicated (in the presence of SSA) and IL-6 (panel A) and IFNβ (panel B) mRNA levels were monitored by qPCR. mRNA levels were normalized so that 1 = mRNA levels in the absence of stimulation. Asterisks indicate values that are significantly different from control treatment in the presence of LPS (p<0.05).
(PDF)

Figure S7 Inhibition of Psmd3 does not alter MyD88 mRNA splicing. RAW264.7 cells were transfected with the indicated siRNAs (*Psmd3* or control nontargeting siRNA “CT”). The cells were then stimulated with LPS (20 ng/ml for six hours) and either IL-6 production was monitored by ELISA (panel A) or MyD88_L_ and MyD88_S_ production was monitored by qPCR (panels B and C). Asterisks indicate values that are significantly different from control treatment in the presence of LPS (p<0.05).
(PDF)

Figure S8 Longer exposure of agarose gel in Figure 5D. Differences in MyD88_S_ are more clear in this longer exposure.
(PDF)

Figure S9 Gene knockdown data for Figure 6C. Cells were transfected with the indicated siRNAs and knockdown of *Sf3a1* (panel A), *MyD88_L_* (panel B), or *MyD88_S_* (panel C) were measured by qPCR as indicated. Asterisks indicate values that are significantly different from control treatment (p<0.05).
(PDF)

Table S1 Oligonucleotides used for qPCR and RT-PCR.
(PDF)
