# Supplementary figures and images for: Limiting of the Innate Immune Response by SF3A-Dependent Control of MyD88 Alternative mRNA Splicing
Source: PLoS Genet. 2013 Oct 24;9(10):e1003855. doi: 10.1371/journal.pgen.1003855 (PMC3812059; doi:10.1371/journal.pgen.1003855)

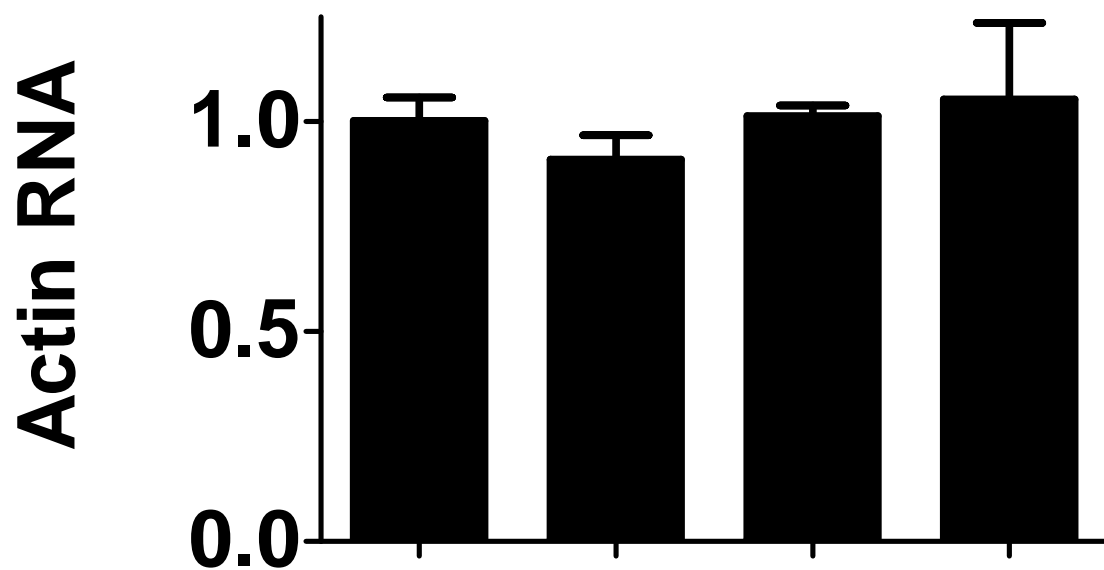

CT siRNA: + + - -

SF3a1 siRNA: - - + +

LPS: - + - +

Supplement: Figure S1 — Inhibition of SF3A1 does not alter excision of βactin intron 3. RAW264.7 cells were transfected with either Sf3a1 siRNA or control non-targeting siRNA (CT) and were subsequently stimulated with LPS (20 ng/ml for 6 hr) or not as indicated. Cells were then lysed and qPCR was used to monitor βactin mRNA levels. The figure depicts mRNA levels for primers that both annealed to exon 4 normalized relative to primers that annealed to exons 3 and 4 and therefore span intron 3. No significant difference was observed between the two primer sets. (PDF) [file pgen.1003855.s001.pdf]

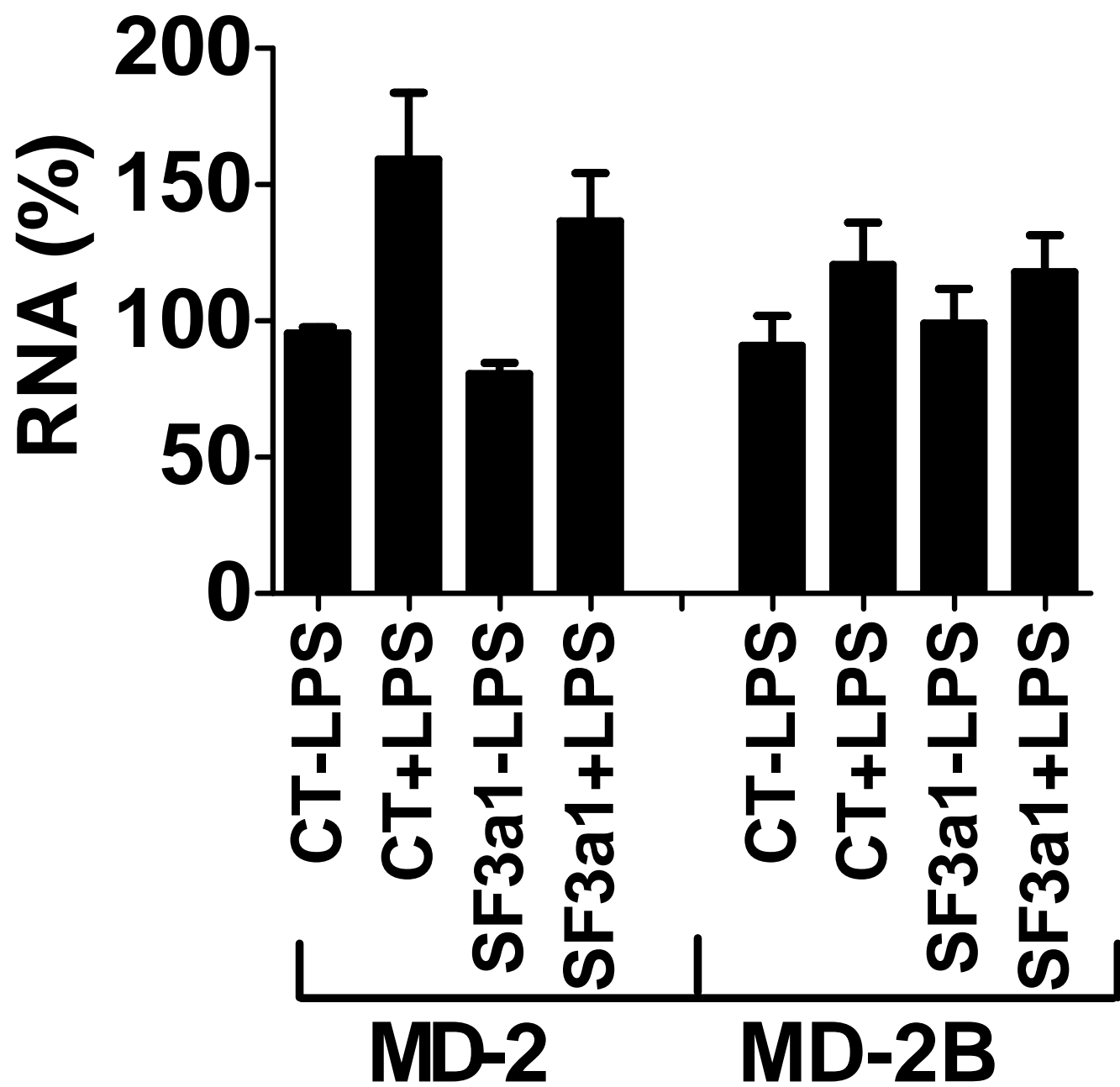

Supplement: Figure S2 — Inhibition of SF3A1 does not alter the alternative splicing of MD-2 mRNA. RAW264.7 cells were transfected with either SF3a1 siRNA or control non-targeting siRNA (CT) and were subsequently stimulated with LPS (20 ng/ml for 6 hr) or not as indicated. Cells were then lysed and qPCR was used to monitor production of MD-2 or MD-2B mRNA. No significant difference was observed for either isoform. (PDF) [file pgen.1003855.s002.pdf]

**A**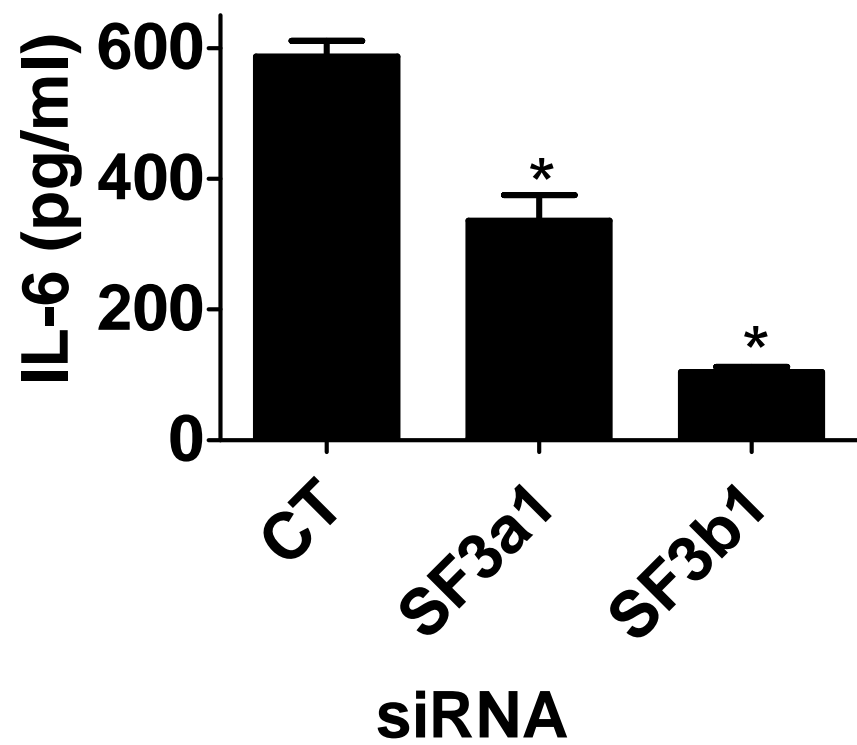**B**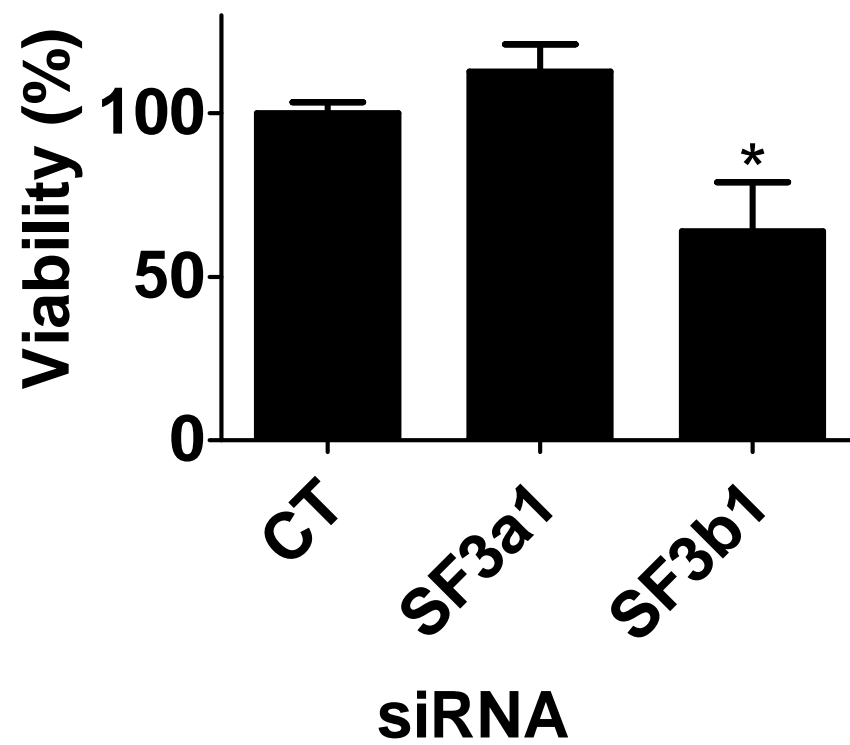

Supplement: Figure S3 — Inhibition of SF3A1 or SF3B1 in differentiated human THP1 macrophages diminishes LPS-induced IL-6 production. The human monocyte cell line THP1 was transfected with the indicated siRNAs (Sf3a1, Sf3b1, or control non-targeting siRNA “CT”). The cells were then differentiated with PMA, exposed to LPS for six hours (50 ng/ml), and then IL-6 (panel A) and viability (panel B) were monitored. Asterisks indicate values that are significantly different from control treatment (p<0.05). (PDF) [file pgen.1003855.s003.pdf]

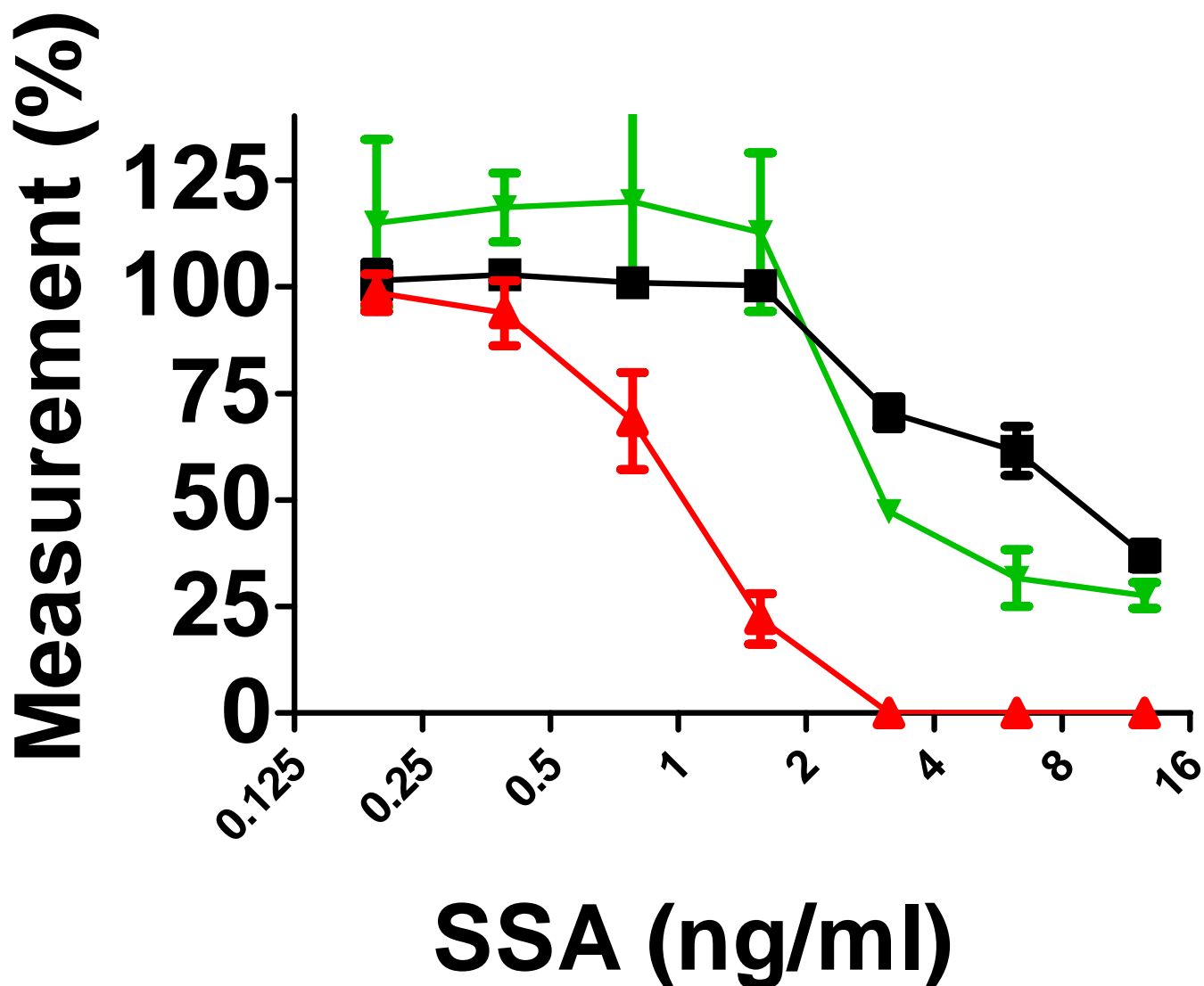

- Viability (%)
- ▼ Phagocytosis (%)
- ▲ IL-6 (%)

Supplement: Figure S4 — Treatment of cells with spliceostatin A (SSA) inhibits LPS-induced IL-6 production. The figure depicts a dose-response titration of SSA at the indicated doses. Cells were treated with SSA for six hours, were subsequently exposed to 20 ng/ml LPS for an additional six hours (in the presence of SSA), and then the cells were monitored for either viability, phagocytosis of FITC-labeled E. coli particles, or production of IL-6. All values are normalized relative to control cells not treated with SSA. At high doses, SSA diminishes all three readouts. However, at lower doses, SSA inhibits IL-6 production without significantly affecting viability or phagocytosis. (PDF) [file pgen.1003855.s004.pdf]

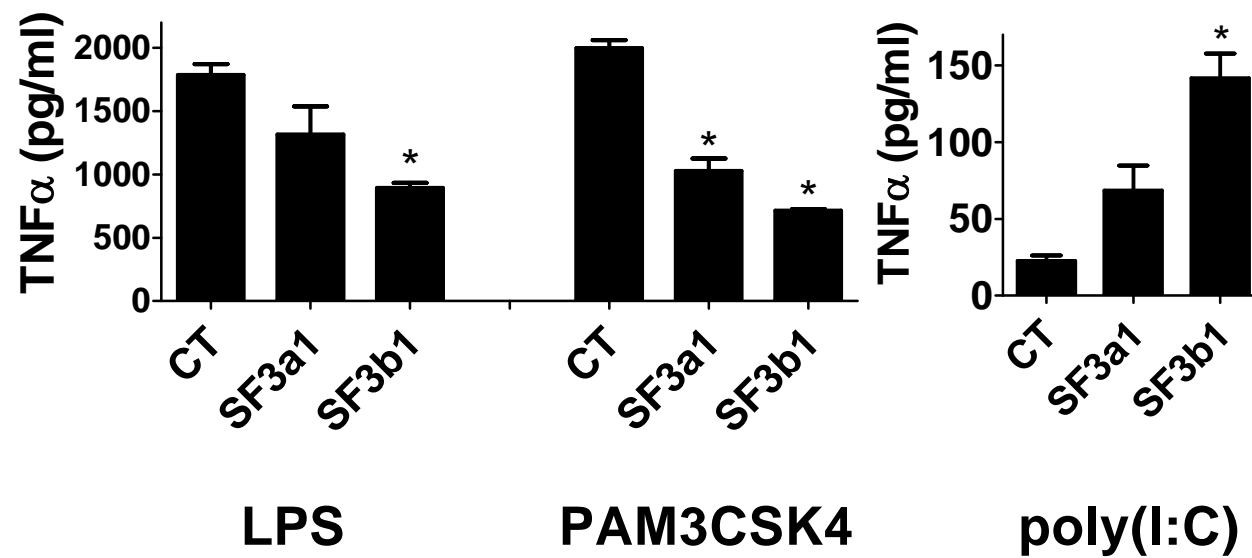

Supplement: Figure S5 — Inhibition of SF3A1 or SF3B1 weakens the response to TLR4 and TLR2/1 agonists but not the response to TLR3 agonists. RAW264.7 cells were transfected with the indicated siRNAs (SF3a1, SF3b1, or control nontargeting siRNA “CT”). The cells were then stimulated with either 20 ng/ml LPS, 1.5 µg/ml PAM3CSK4, or 6 µg/ml poly(I:C) for six hours, and then TNFα production was monitored by ELISA. Asterisks indicate values that are significantly different from control treatment (p<0.05). (PDF) [file pgen.1003855.s005.pdf]

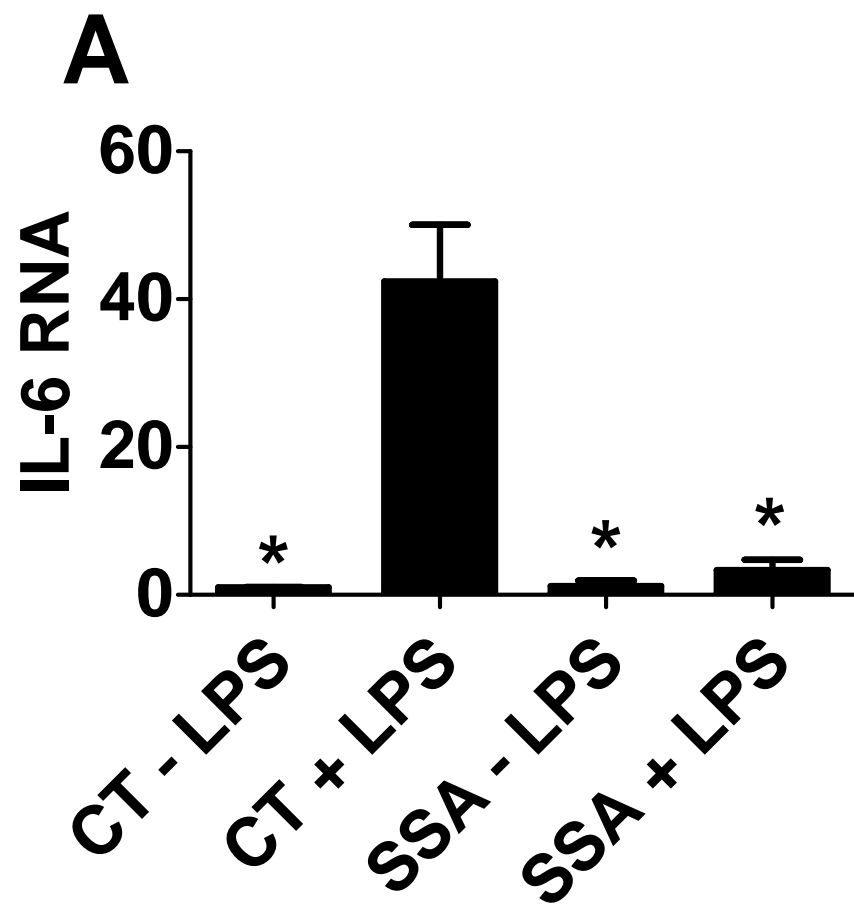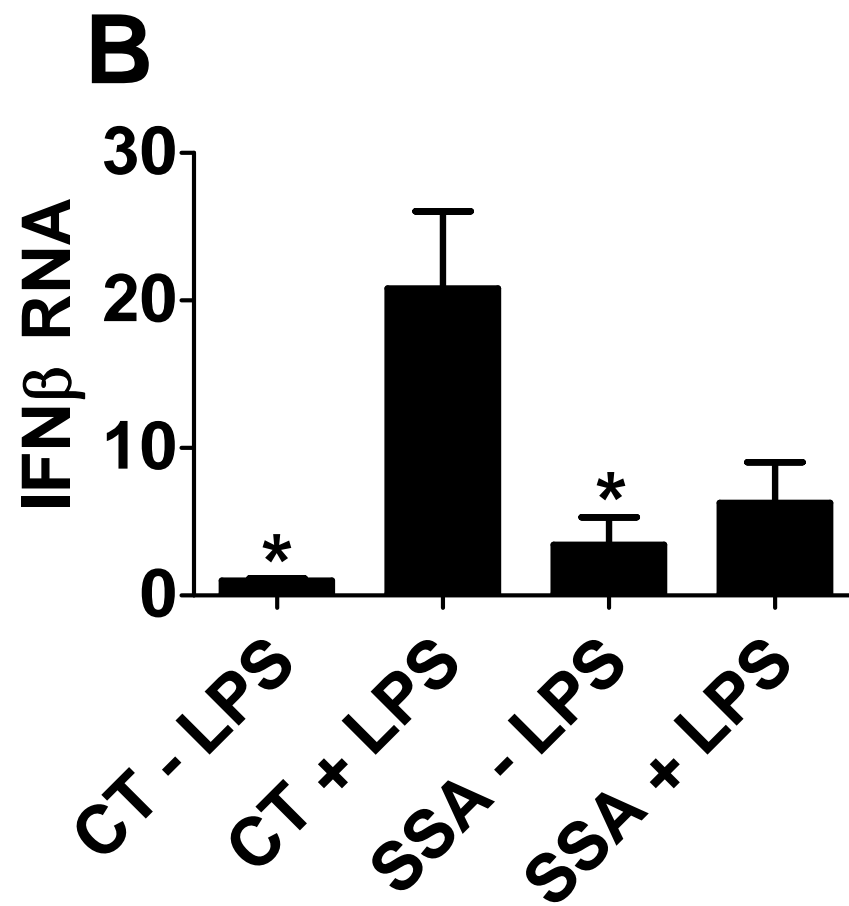

Supplement: Figure S6 — SSA treatment inhibits LPS-induced production of IL-6 and IFNβ. RAW264.7 cells were treated with 1.56 ng/ml SSA for 12 hours. The cells were then stimulated with 20 ng/ml LPS for six hours where indicated (in the presence of SSA) and IL-6 (panel A) and IFNβ (panel B) mRNA levels were monitored by qPCR. mRNA levels were normalized so that 1 = mRNA levels in the absence of stimulation. Asterisks indicate values that are significantly different from control treatment in the presence of LPS (p<0.05). (PDF) [file pgen.1003855.s006.pdf]

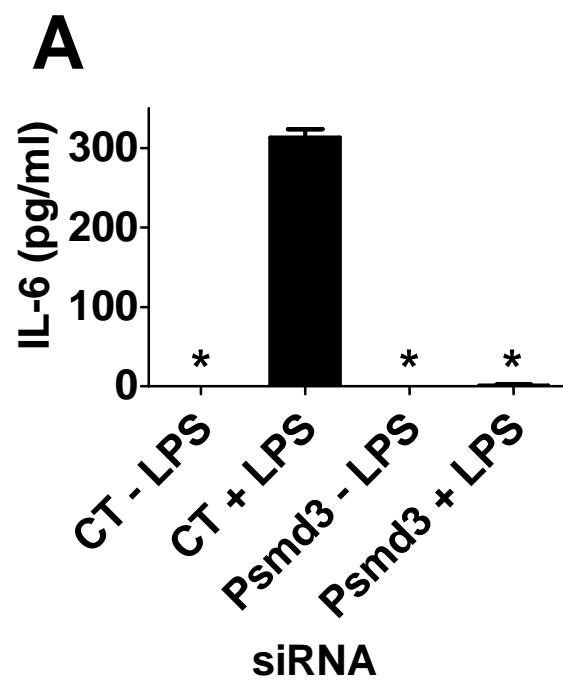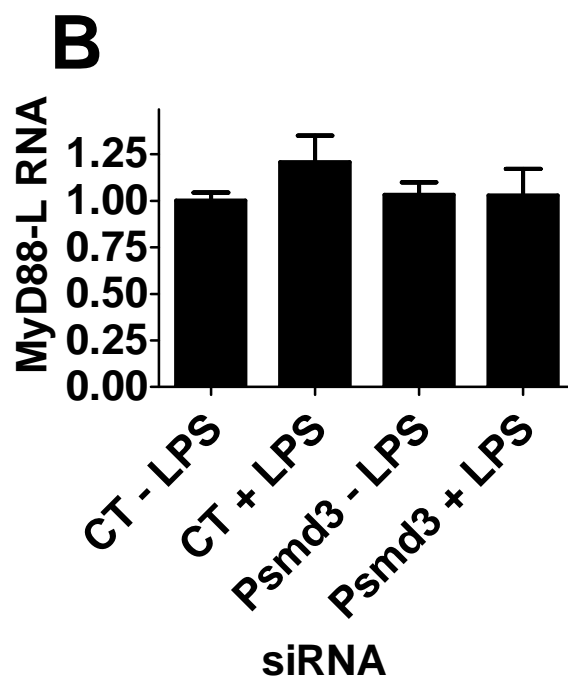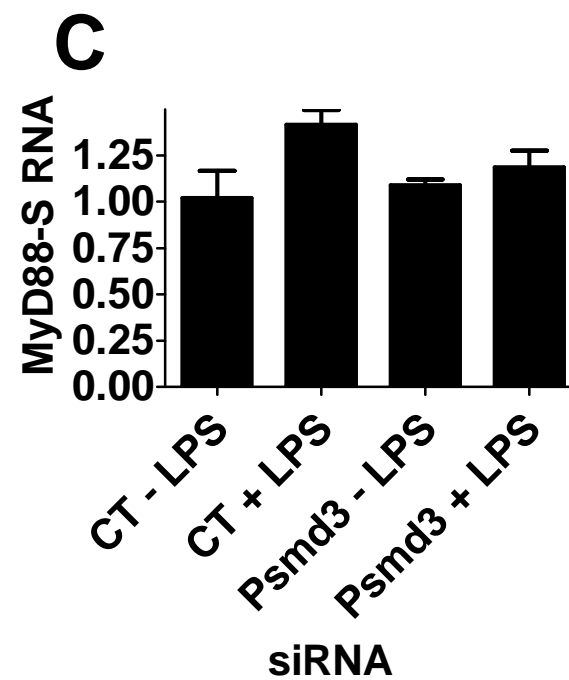

Supplement: Figure S7 — Inhibition of Psmd3 does not alter MyD88 mRNA splicing. RAW264.7 cells were transfected with the indicated siRNAs (Psmd3 or control nontargeting siRNA “CT”). The cells were then stimulated with LPS (20 ng/ml for six hours) and either IL-6 production was monitored by ELISA (panel A) or MyD88L and MyD88S production was monitored by qPCR (panels B and C). Asterisks indicate values that are significantly different from control treatment in the presence of LPS (p<0.05). (PDF) [file pgen.1003855.s007.pdf]

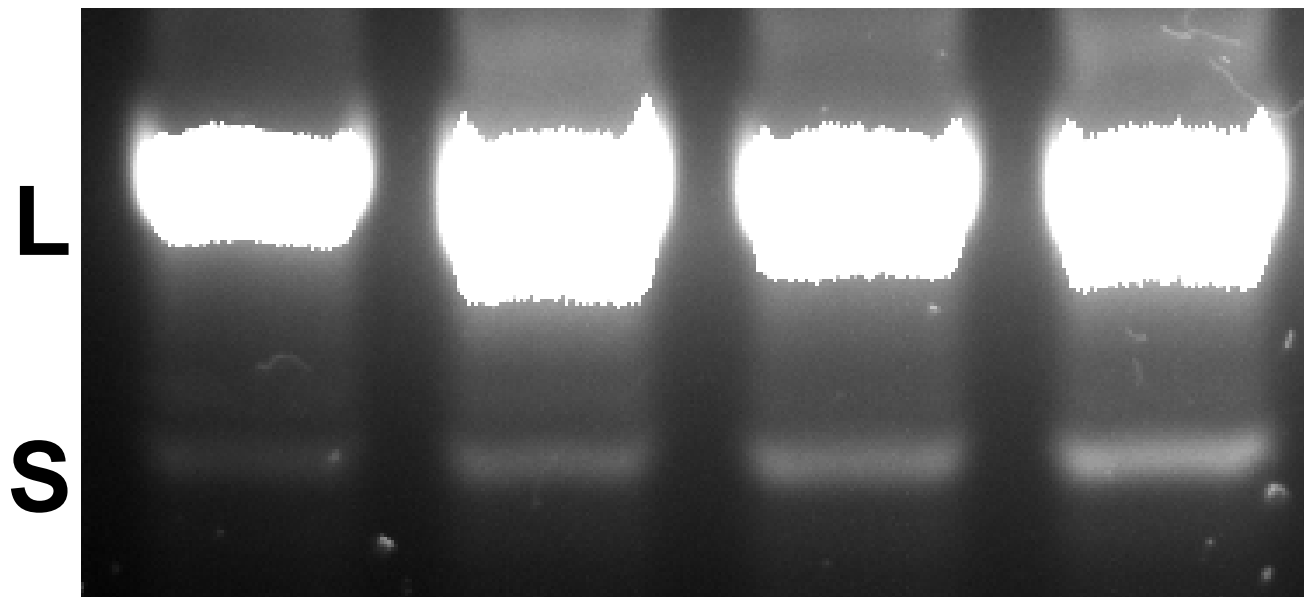

|        |    |    |       |       |
|--------|----|----|-------|-------|
| siRNA: | CT | CT | Sf3a1 | Sf3a1 |
| LPS:   | -  | +  | -     | +     |

Supplement: Figure S8 — Longer exposure of agarose gel in Figure 5D. Differences in MyD88S are more clear in this longer exposure. (PDF) [file pgen.1003855.s008.pdf]

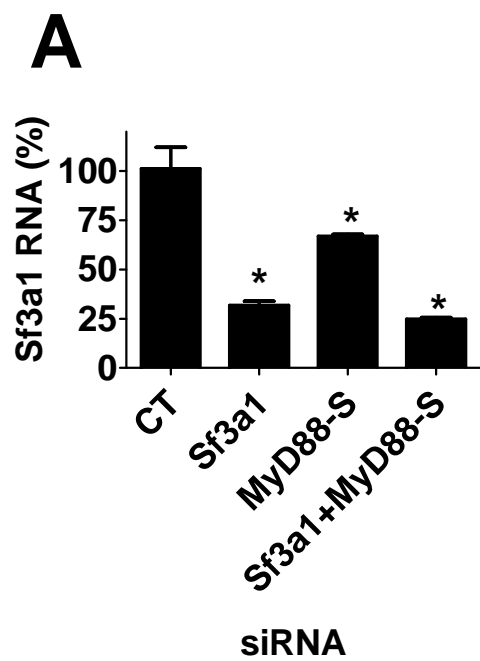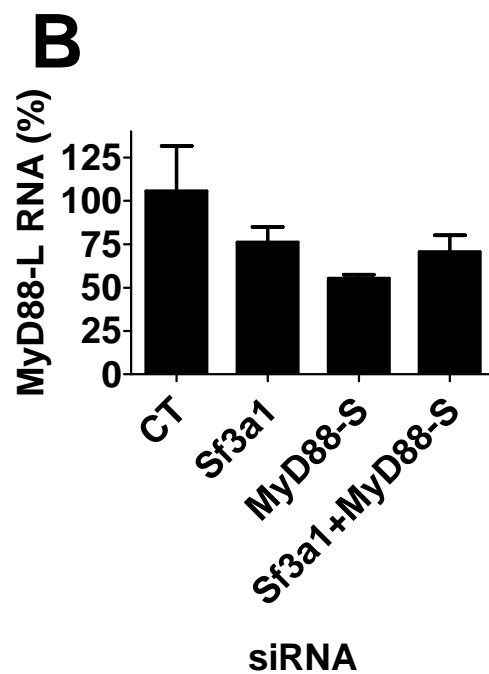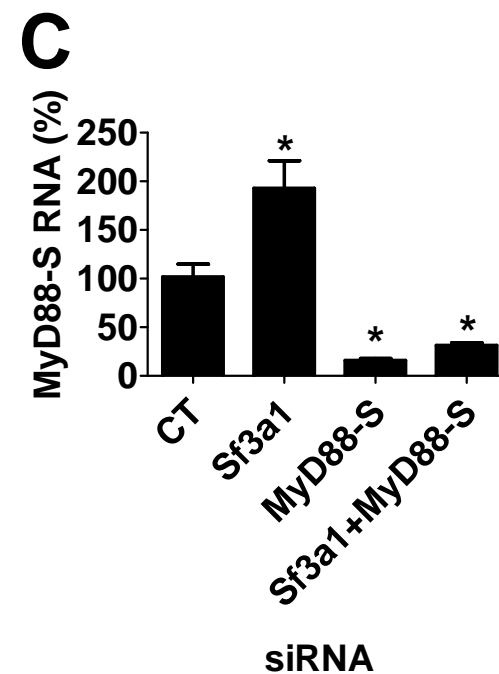

Supplement: Figure S9 — Gene knockdown data for Figure 6C. Cells were transfected with the indicated siRNAs and knockdown of Sf3a1 (panel A), MyD88L (panel B), or MyD88S (panel C) were measured by qPCR as indicated. Asterisks indicate values that are significantly different from control treatment (p<0.05). (PDF) [file pgen.1003855.s009.pdf]
