# Supplementary material for: Limiting of the Innate Immune Response by SF3A-Dependent Control of MyD88 Alternative mRNA Splicing
Source: PLoS Genet. 2013 Oct 24;9(10):e1003855. doi: 10.1371/journal.pgen.1003855 (PMC3812059; doi:10.1371/journal.pgen.1003855)
Supplement: Table S1 — Oligonucleotides used for qPCR and RT-PCR. (PDF) [file pgen.1003855.s010.pdf]

Table S1. Oligonucleotides used for qPCR and RT-PCR

Gene	Left Primer		Right Primer		Notes
	**Name**	**Sequence**	**Name**	**Sequence**	
beta actin	oSA664	CTGGCCGGGACCTGACAGACTACCTCATG	oSA665	GATGTCACGCACGATTTCCCTCTCAGCTG	Both in exon 4; qPCR & RT-PCR
beta actin	mBactin-f	CTGAACCCTAAGGCCAACCGTG	mBactin-r	CCGTCTCCGGAGTCCATCACAATG	Brackets intron 3; qPCR & RT-PCR
IL-6	IL-6 fwd	CTTGGGACTGATGCTGGTGAC	IL-6 rev	GCCTCCGACTTGTGAAGTGGTATAGACAGG	qPCR
IFNbeta	IFNb-qPCR-F	GGCTTCCATCATGAACAACAGGTGGATCC	IFNb-qPCR-R	CCAGGAGCTCCTGACATTTCCGAATGTTCGTCC	qPCR
MD-2	oSA777	TTGTGCATGTTGAGTTCATTCCAAGAGGAAAC	oSA778	CCTTACGCTTCGGCAACTCTATGGAGTTGAC	qPCR
MD-2B	oSA781	GATTTGTGCATGTTGAGTTCATTCCAAAGTTGCC	oSA780	CCCTCGAAAGAGAATGGTATTGATGTATTCACAGTC	qPCR
MyD88-L	oSA656	CCACCCTTGATGACCCCTAGGACAAAC	oSA657	GTCTGTTCTAGTTGCCGGATCATCTCCTGCAC	qPCR
MyD88-S	oSA658	GGAGCTGAAGTCGCGCATCGGACAAAC	oSA657	GTCTGTTCTAGTTGCCGGATCATCTCCTGCAC	qPCR
MyD88-S	oSA666	TTGTTGGATGCCTGGCAGGGGCGCTCTGGC	oSA659	GTTCCGGCGTTTGTCCGATGC	RT-PCR
MyD88-L/S	oSA666	TTGTTGGATGCCTGGCAGGGGCGCTCTGGC	oSA669	CACGGTCGGACACACACAACTTAAGCCGATAGTC	RT-PCR
Sf3a1	oSA620	GCAGCAGCAAGCTACGCAACAGCAGCTGCC	oSA621	GCG ATG AACTCA AACTCA GGA GGG GGCTCC	qPCR
Sf3a2	oSA624	CTGCGCCAATTGGCCCTGGAAACCATTGACATC	oSA625	CTG ATG TTT CTT CCCTTG GGT ATG GGC CAG GTA GC	qPCR
Sf3a3	oSA628	GTGGGAACCTGAGGGATTTGTATGATGATAAGGAC	oSA629	GAATTC AGC GAA CTC GTT GGGTCC TGA GAT AGC	qPCR
Sf3b1	Sf3b1-l1	CCATGATAATTTCCCCAGAGCGTCTTGATCC	Sf3b1-r1	GTT CTC GCATAA CAT CCATGT AGGTTC TAG CAT TC	qPCR
oligo-dT	oligo-dT	from Applied Biosystems			RT
MyD88 RT	oSA667	GCTGTCCCAAAGGAAACACACATATGCAGATGCC			RT
